# Supplementary material for: Cytocipher determines significantly different populations of cells in single-cell RNA-seq data
Source: Bioinformatics. 2023 Jul 14;39(7):btad435. doi: 10.1093/bioinformatics/btad435 (PMC10368802; doi:10.1093/bioinformatics/btad435)
Supplement: btad435_Supplementary_Data [file btad435_supplementary_data.pdf]

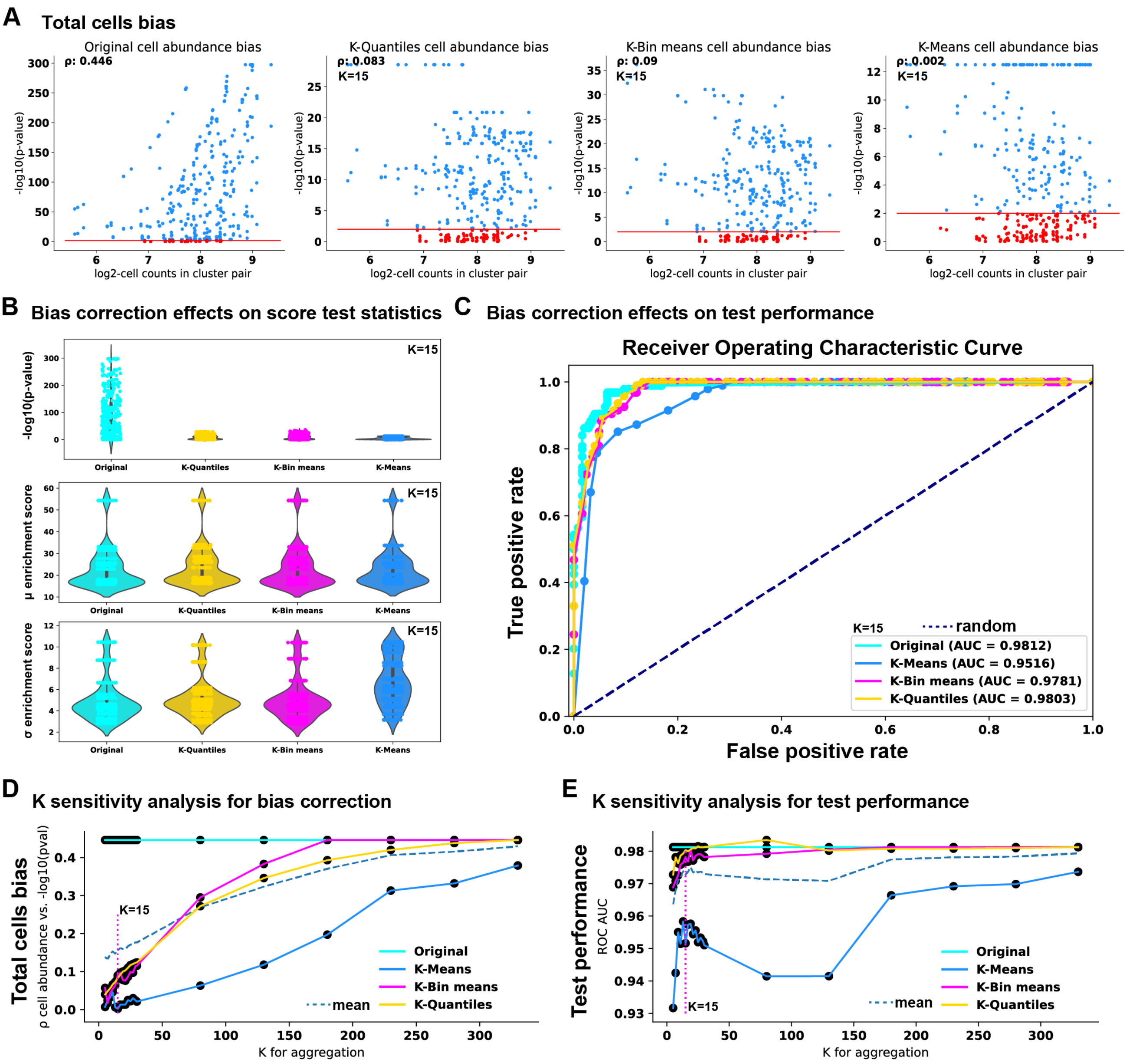

Fig. 1: **Supplementary** *Cytocipher code-score* summarisation prior to testing corrects significance bias toward higher cell abundance while maintaining high test performance. **A.** Scatter plots depicting -log10(p-value) and log2 of total cell counts in each cluster pair using over-clustered (Leiden resolution 2.0) peripheral blood mononuclear cells (PBMCs). Each individual point represents a pair of clusters which were compared for significant differences using code-scores. Red points are significantly different cluster pairs at p=0.01, with the red vertical line indicating this cutoff. ρ depicts Spearman's correlation for cluster pair significance and the total number of cells in each cluster (the y- and x- axes, respectively). Separate scatter plots represent the same test applied with different methods for summarising enrichment scores prior to significance testing. From left to right, no summarisation (original), K-Quantiles summarisation, K-Bins summarisation, and summarisation by K-Means are shown. K=15 was used in each case. Each method successfully reduced the correlation of cluster pair significance (-log10(p-values)) and the total number of cells. **B.** Violin plots depict the effect of summarisation methods on key test statistics; -log10(p-values) and the mean/standard deviations of code enrichment scores within each cluster. All methods reduced the p-value inflation while maintaining the mean and standard deviation of the enrichment scores, with the exception of K-Means which inflated the standard deviations. **C.** Receiver operating characteristic (ROC) curve benchmarking each method. Legend indicates area under the curve (AUC) scores (1 indicates perfect classification). K-Quantiles had the best test performance (AUC=0.9803), prompting this as the default summarisation method prior to *Cytocipher* cluster pair significance testing. **D.** Line plot displaying sensitivity analysis when varying the K-parameter and measuring the effect on cell abundance bias. Magenta dotted line indicates K=15 value. Cell abundance bias increased with k. E. Line plot for K-parameter sensitivity with respect to ROC AUC scores. Test performance was marginally affected in the range k>15. k<15 resulted in a drop in performance. This prompted k=15 as the default.

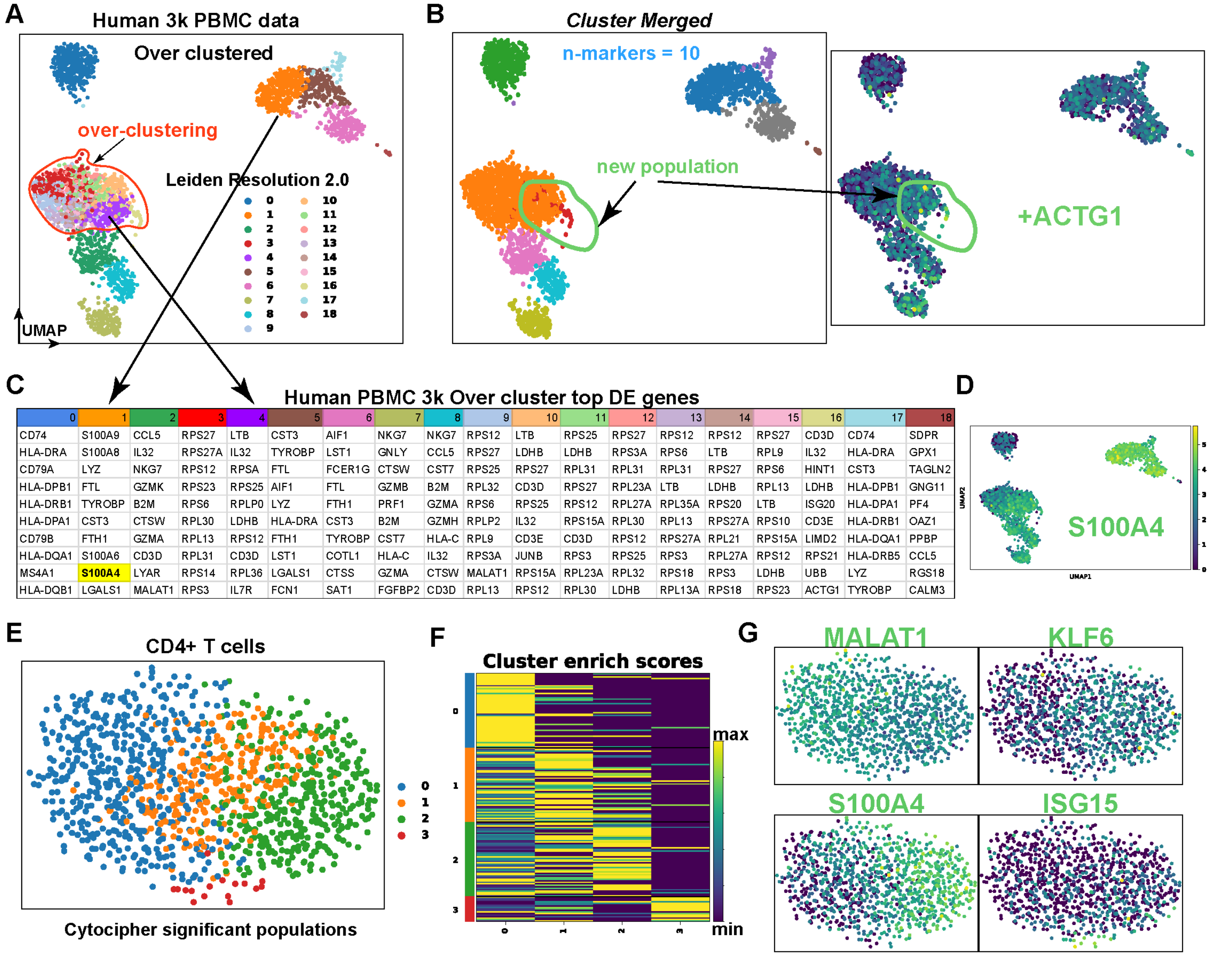

0	1	2	3	4	5	6	7	8	9	10	11	12	13	14	15	16	17	18
CD74	S100A9	CCL5	RPS27	LTB	CST3	AIF1	NKG7	NKG7	RPS12	LTB	RPS25	RPS27	RPS12	RPS12	RPS27	CD3D	CD74	SDPR
HLA-DRA	S100A8	IL32	RPS27A	IL32	TYROBP	LST1	GNLY	CCL5	RPS27	LDHB	LDHB	RPS3A	RPS6	LTB	RPL9	IL32	HLA-DRA	GPX1
CD79A	LYZ	NKG7	RPS12	RPSA	FTL	FCER1G	CTSW	CST7	RPS25	RPS27	RPL31	RPL31	RPL31	RPS27	RPS6	HINT1	CST3	TAGLN2
HLA-DPB1	FTL	GZMK	RPS23	RPS25	AIF1	FTL	GZMB	B2M	RPL32	CD3D	RPS27	RPL23A	LTB	LDHB	RPL13	LDHB	HLA-DPB1	GNG11
HLA-DRB1	TYROBP	B2M	RPS6	RPLP0	LYZ	FTH1	PRF1	GZMA	RPS6	RPS25	RPS12	RPL27A	RPL35A	RPS20	LTB	ISG20	HLA-DPA1	PF4
HLA-DPA1	CST3	CTSW	RPL30	LDHB	HLA-DRA	CST3	B2M	GZMH	RPLP2	IL32	RPS15A	RPL30	RPL13	RPS27A	RPS10	CD3E	HLA-DRB1	OAZ1
CD79B	FTH1	GZMA	RPL13	RPS12	FTH1	TYROBP	CST7	HLA-C	RPL9	CD3E	CD3D	RPS12	RPS27A	RPL21	RPS15A	LIMD2	HLA-DQA1	PPBP
HLA-DQA1	S100A6	CD3D	RPL31	CD3D	LST1	COTL1	HLA-C	IL32	RPS3A	JUNB	RPS3	RPS25	RPS3	RPL27A	RPS12	RPS21	HLA-DRB5	CCL5
MS4A1	**S100A4**	LYAR	RPS14	RPL36	LGALS1	CTSS	GZMA	CTSW	MALAT1	RPS15A	RPL23A	RPL32	RPS18	RPS3	LDHB	UBB	LYZ	RGS18
HLA-DQB1	LGALS1	MALAT1	RPS3	IL7R	FCN1	SAT1	FGFBP2	CD3D	RPL13	RPS12	RPL30	LDHB	RPL13A	RPS18	RPS23	ACTG1	TYROBP	CALM3

Fig. 2: **Supplementary** *CD4+ naive and CD4+ memory T cells are not distinguished by Cytocipher when analysing all PBMC cell types, but can be distinguished when analysing CD4+ T cells in isolation.* **A.** Over-clustered human 3K PBMC data. **B.** Additional population of CD4+ T cells found by increasing marker genes in *Cytocipher* to 10. This population has higher expression of ACTG1, which is a marker for recently activated CD4+ T cells (Lyu et al., 2021). **C.** Top 10 marker genes for each PBMC over clusters. CD4+ naive versus CD4+ memory T cell markers are not apparent in the top DE genes for the CD4+ over-clusters (CD4+ memory markers: S100A4 (Weatherly et al., 2015), CCR5 (Martín-Leal et al., 2020), CXCR3 (Groom and Luster, 2011), CD69 (Schoenberger, 2012), CD57 (Ahmed et al., 2020); CD4+ naive markers: CD62L (Pakpour et al., 2008), TCF1 (Escobar et al., 2020)). **D.** Expression of S100A4 is apparent as a marker gene for monocyte and dendritic cell over-clusters. S100A4 was not distinguished as a marker gene for T cell over-clusters due to broad expression in other cell types, and was not therefore used to distinguish CD4+ naive versus memory T-cells by *Cytocipher*. **E.** UMAP of isolated CD4+ T cells, coloured by significant populations determined by *Cytocipher*, illustrates additional T cell populations can be identified after subsetting. **F.** *Cytocipher code-scores* for cells in each significant population. **G.** Marker genes for each of the CD4+ populations determined by *Cytocipher*. Naive versus memory CD4+ T cells are distinguishable by S100A4 expression using *Cytocipher* after subsetting to CD4+ T cells. Intermediate T cells between naive and memory CD4+ T cells are also apparent, along with an additional T cell population specifically expressing ISG15. Overall, this illustrates additional cell populations may be distinguished by *Cytocipher* when analysing subsets of cells.

References

Raya Ahmed, Kelly L. Miners, Julio Lahoz-Beneytez, Rhiannon E. Jones, Laureline Roger, Christina Baboonian, Yan Zhang, Eddie C.Y. Wang, Marc K. Hellerstein, Joseph M. McCune, Duncan M. Baird, David A. Price, Derek C. Macallan, Becca Asquith, and Kristin Ladell. Cd57+ memory t cells proliferate in vivo. *Cell Reports*, 33(11):108501, 2020. ISSN 2211-1247. doi: https://doi.org/10.1016/j.celrep.2020.108501.

Giulia Escobar, Davide Mangani, and Ana C. Anderson. T cell factor 1: A master regulator of the t cell response in disease. *Science Immunology*, 5(53):eabb9726, 2020. doi: 10.1126/sciimmunol.abb9726.

Joanna R. Groom and Andrew D. Luster. Cxcr3 in t cell function. *Experimental Cell Research*, 317(5):620–631, 2011. ISSN 0014-4827. doi: https://doi.org/10.1016/j.yexcr.2010.12.017. Special Issue: Chemokines.

Menghua Lyu, Shiyu Wang, Kai Gao, Longlong Wang, Xijun Zhu, Ya Liu, Meiniang Wang, Xiao Liu, Bin Li, and Lei Tian. Dissecting the landscape of activated cmv-stimulated cd4+ t cells in humans by linking single-cell rna-seq with t-cell receptor sequencing. *Frontiers in Immunology*, 12, 2021. ISSN 1664-3224. doi: 10.3389/fimmu.2021.779961.

Ana Martín-Leal, Raquel Blanco, Josefina Casas, María E Sáez, Elena Rodríguez-Bovolenta, Itziar de Rojas, Carina Drechsler, Luis Miguel Real, Gemma Fabrias, Agustín Ruíz, Mario Castro, Wolfgang WA Schamel, Balbino Alarcón, Hisse M van Santen, and Santos Mañes. Ccr5 deficiency impairs cd4+ t-cell memory responses and antigenic sensitivity through increased ceramide synthesis. *The EMBO Journal*, 39(15):e104749, 2020. doi: https://doi.org/10.15252/embj.2020104749.

Nazzy Pakpour, Colby Zaph, and Phillip Scott. The Central Memory CD4+ T Cell Population Generated during Leishmania major Infection Requires IL-12 to Produce IFN-1. *The Journal of Immunology*, 180(12):8299–8305, 06 2008. ISSN 0022-1767. doi: 10.4049/jimmunol.180.12.8299.

Stephen P. Schoenberger. Cd69 guides cd4¡sup¿+¡/sup¿ t cells to the seat of memory. *Proceedings of the National Academy of Sciences*, 109(22):8358–8359, 2012. doi: 10.1073/pnas.1204616109.

Kathleen Weatherly, Marie Bettonville, David Torres, Arnaud Kohler, Stanislas Goriely, and Michel Y. Braun. Functional profile of s100a4-deficient t cells. *Immunity, Inflammation and Disease*, 3(4):431–444, 2015. doi: https://doi.org/10.1002/iid3.85.
